# The Neuroprotective Role of Protein Quality Control in Halting the Development of Alpha-Synuclein Pathology

**DOI:** 10.3389/fnmol.2017.00311

**Published:** 2017-09-27

**Authors:** Destiny-Love Manecka, Benoît Vanderperre, Edward A. Fon, Thomas M. Durcan

**Affiliations:** Neurodegenerative Diseases Group and iPSC-CRISPR Core Facility, Montreal Neurological Institute, McGill University, Montreal, QC, Canada

**Keywords:** α-synuclein, protein quality control, Lewy body, Parkinson’s disease, chaperone, autophagy, lysosome

## Abstract

Synucleinopathies are a family of neurodegenerative disorders that comprises Parkinson’s disease, dementia with Lewy bodies, and multiple system atrophy. Each of these disorders is characterized by devastating motor, cognitive, and autonomic consequences. Current treatments for synucleinopathies are not curative and are limited to improvement of quality of life for affected individuals. Although the underlying causes of these diseases are unknown, a shared pathological hallmark is the presence of proteinaceous inclusions containing the α-synuclein (α-syn) protein in brain tissue. In the past few years, it has been proposed that these inclusions arise from the self-templated, prion-like spreading of misfolded and aggregated forms of α-syn throughout the brain, leading to neuronal dysfunction and death. In this review, we describe how impaired protein homeostasis is a prominent factor in the α-syn aggregation cascade, with alterations in protein quality control (PQC) pathways observed in the brains of patients. We discuss how PQC modulates α-syn accumulation, misfolding and aggregation primarily through chaperoning activity, proteasomal degradation, and lysosome-mediated degradation. Finally, we provide an overview of experimental data indicating that targeting PQC pathways is a promising avenue to explore in the design of novel neuroprotective approaches that could impede the spreading of α-syn pathology and thus provide a curative treatment for synucleinopathies.

## Introduction

**FIGURE 1:**
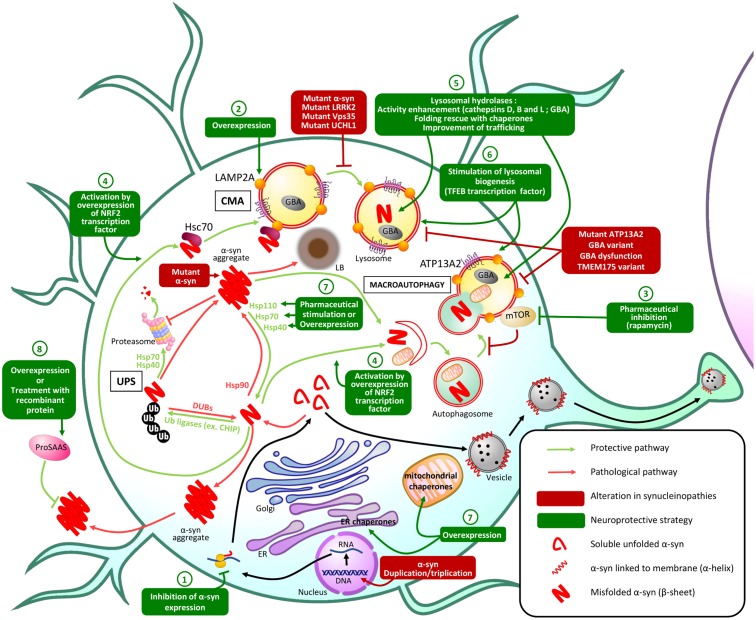
Principal PQC mechanisms involved in α-syn homeostasis and potential therapeutic approaches. In physiologic conditions, misfolded α-syn protein is degraded by PQC machinery: the UPS is responsible for ubiquitination of α-syn leading to proteasomal degradation of α-syn, while macroautophagy and CMA both lead to lysosomal degradation of misfolded α-syn. In synucleinopathies, alterations in these protective mechanisms result in the accumulation of misfolded α-syn in aggregates and Lewy bodies (LB) that lead to neuronal dysfunction and death. Genetic or pharmaceutical approaches to restore altered PQC pathways or stimulate alternative PQC pathways: (1) Inhibition of α-syn expression could prevent its pathological accumulation. (2) Overexpression of Lamp2A lysosomal receptor could increase the CMA of misfolded α-syn. (3) Pharmaceutical inhibition of mTOR, an autophagy receptor, stimulates macroautophagy preventing α-syn accumulation and aggregation. (4) Overexpression of the transcription factor NRF2 activates both macroautophagy and CMA and stimulates the lysosomal degradation of misfolded α-syn. (5) Improving lysosomal hydrolases activity or (6) stimulating lysosomal biogenesis could also enhance α-syn lysosomal degradation. α-syn aggregation can also be prevented by stimulation or overexpression of (7) endogenous or (8) secretory chaperones.

Maintaining protein homeostasis is essential for normal cellular function and viability. This is overseen by PQC mechanisms, through the control of protein synthesis, localization, folding/refolding, degradation and formation of protein inclusions. At the post-translational level, PQC is orchestrated by several mechanisms including chaperones that maintain correct protein conformation or help refold misfolded proteins; and the UPS and ALP, which degrade proteins that are irreversibly misfolded, damaged, or are no longer required by the cell. In this review, we focus on these aspects of PQC; with other PQC pathways reviewed elsewhere (Wolff et al., 2014; Dubnikov et al., 2017). In eukaryotes, protein chaperones are essential for ensuring the correct folding of nascent proteins and refolding of misfolded proteins. Hsps or heat shock chaperones (Hscs) are a prominent group of chaperones and they can be found in the ER, mitochondria, cytoplasm or extracellular space (Hartl et al., 2011; Wyatt et al., 2013). Protein degradation through the UPS is regulated by the sequential activity of E1, E2, and E3 enzymes that conjugate primarily K48-linked ubiquitin (Ub) chains onto lysine residues in proteins destined for elimination through the 26S proteasome (Passmore and Barford, 2004). The ALP acts mainly through macroautophagy and CMA. In macroautophagy, cytoplasmic content (including soluble and aggregated proteins) is engulfed by a double-membrane to form an autophagosome that fuses with the lysosome forming an autolysosome, degrading the autophagosomal content (Bento et al., 2016). In CMA, Hsc70 specifically binds to and targets proteins containing KFERQ-like motifs to the lysosomal receptor Lamp2A for client import through the lysosomal membrane, and subsequent degradation by lysosomal hydrolases (Cuervo and Wong, 2014). In addition, chaperones and ubiquitination systems promote the spatial sequestration of misfolded proteins into inclusions (aggresome/Q-bodies) and mediate the lysosomal degradation of toxic aggregates through the aggresome–autophagy and multivesicular body pathways (Johnston et al., 1998; Sahu et al., 2011; Escusa-Toret et al., 2013; Sontag et al., 2017). These pathways are regulated by K63-linked Ub chains and are critical for the degradation of aggregated proteins including α-synuclein (α-syn) (Tanaka et al., 2004; Filimonenko et al., 2007; Tofaris et al., 2011). Inefficient PQC is implicated in protein toxicity, gain- or loss-of-function in many pathologies, including several neurodegenerative diseases known as synucleinopathies. Synucleinopathies, which include PD, LBD and MSA, are characterized by the pathologic accumulation and aggregation of α-syn (McCann et al., 2014). As some mutations altering PQC machinery are associated with familial forms of synucleinopathies and α-syn pathologic aggregates impair PQC, targeting the PQC machinery has become a promising therapeutic strategy for opposing the toxic effects of misfolded α-syn aggregates (**Figure 1**).

## Misfolding and Spreading of α-syn in Synucleinopathies

α-syn, a 140-amino acid protein encoded by the *SNCA* gene, is abundant in the human brain (1% of all cytosolic proteins) (Stefanis, 2012) and is implicated in various cellular processes including vesicular trafficking, dopamine release and reuptake (Abeliovich et al., 2000; Senior et al., 2008; DeWitt and Rhoades, 2013; Burré et al., 2014). α-syn is structured into three domains: the N-terminal amphipathic domain, which allows membrane-binding; the central hydrophobic non-amyloid-β component (NAC) domain, essential for α-syn aggregation; and the acidic, negatively charged C-terminal domain, that is critical for chaperone-like activity during thermal and chemical stress (Souza et al., 2000; Ahn et al., 2006; Beyer, 2006). Under physiological conditions, α-syn is soluble and intrinsically disordered, or can adopt an N-terminal α-helix conformation with high affinity for biological membranes (Bartels et al., 2011). In synucleinopathies, α-syn follows sequential aggregation/fibrillization, starting from soluble monomers, dimers, and misfolded oligomers that aggregate into insoluble protofibrils and fibrils with an anti-parallel β-sheet structure (Dettmer et al., 2015; Rodriguez et al., 2015; Roeters et al., 2017).

Aggregation of α-syn leads to the formation of proteinaceous inclusions termed Lewy bodies (LB) and Lewy neurites (Wakabayashi et al., 1998). In PD, α-syn pathology has been shown to spread from brainstem to neocortex following a specific pattern (Braak et al., 2003). Recent evidence suggests that this is due to the prion-like, cell-to-cell propagation of α-syn aggregates (Masuda-Suzukake et al., 2013; Goedert et al., 2016). This concept was established in cells containing α-syn fibrils that secrete α-syn seeds taken up by surrounding healthy cells. In these recipient cells, exogenous protofibrils seed the aggregation of endogenous soluble α-syn monomers, causing α-syn to adopt an insoluble β-sheet conformation. This results in the formation of new α-syn seeds, which spread into neighboring cells (Volpicelli-Daley et al., 2011; Luk et al., 2012a,b; Mougenot et al., 2012).

## Targeting PQC Defects as Potential Neuroprotective Strategies Against α-syn Pathology

### Chaperones

The first indication that chaperones confers neuroprotection in α-syn–induced pathogenesis was Hsp70 overexpression protecting against α-syn toxicity in *Drosophila* (Auluck et al., 2002). Accordingly, modulating chaperone function through chemical or genetic approaches holds great therapeutic promise for synucleinopathies. Chaperones ensure the correct folding of nascent and mature protein chains (Ebrahimi-Fakhari et al., 2011; Sharma and Priya, 2017). They also prevent seeding of new aggregates and fibrillization by occluding surfaces that may serve as platforms to induce misfolding of native proteins (Hartl et al., 2011). To some extent, Hsp110, Hsp70 and Hsp40 chaperones can disassemble α-syn fibrillary aggregates *in vitro* (Duennwald et al., 2012; Gao et al., 2015). Enhancing disaggregase activity genetically or with pharmacological modulators could counteract α-syn aggregation (Gao et al., 2015; Jackrel and Shorter, 2015; Shorter, 2016; Sharma and Priya, 2017). Whether disaggregation occurs *in vivo* remains to be established, and since this process might generate soluble, potentially toxic forms of misfolded α-syn, simultaneous enhancement of α-syn degradation is likely necessary for beneficial effects.

Targeting Hsp70/Hsp90 signaling is of prime interest, not only in synucleinopathies, but also in other adult-onset proteinopathies (Pratt et al., 2015). These chaperones have opposing effects: Hsp90 stabilizes its clients, whereas Hsp70 directs them for proteasomal degradation upon Hsp90 dissociation. In yeast, cellular, or animal models of PD, inhibiting Hsp90 activity (Auluck and Bonini, 2002; Auluck et al., 2005; Putcha et al., 2010) or stimulating Hsp70 activity (Auluck et al., 2002; McLean et al., 2002; Klucken et al., 2004; Zhou et al., 2004, 2011; Shin et al., 2005; Yu et al., 2005; Batelli et al., 2008; Outeiro et al., 2008) and that of its collaborator Hsp40 (McLean et al., 2002; Fan et al., 2006) reduces α-syn oligomerization, inclusions formation, and toxicity, and diminishes α-syn levels (**Table 1**). Although induction of chaperone expression in various cellular locations during proteotoxic stress and its associated stress response is observed in the brains of patients affected by synucleinopathies, α-syn aggregates still accumulate, indicating that the chaperone machinery is overwhelmed. This is supported by findings that many chaperones (including Hsp70, Hsp90 and Hsp40) or mediators of the heat-shock response (HDAC6) are found in LBs (**Table 1**), possibly reflecting a cellular attempt to sequester soluble, harmful misfolded species of α-syn (Escusa-Toret et al., 2013). Other chaperones can also mitigate α-syn aggregation and toxicity in various models (see **Table 1**). Overall, it appears evident that modulation of chaperone function is an innovative therapeutic approach against α-syn toxicity. In a clinical context, where widespread α-syn aggregation has already occurred, a global increase in chaperoning activity such as stimulation of the heat-shock response (Du et al., 2014) might have a greater impact than manipulation of individual chaperones. The use of pharmacological chaperones (e.g., flavonoids or polyphenols, Caruana et al., 2011; Ren et al., 2016; Gautam et al., 2017) to prevent or revert α-syn aggregation may also complement therapeutic modulation of endogenous chaperones.

**Table 1:** Therapeutic avenues for targeting PQC pathways in the treatment of synucleinopathies.

Target	Physiological function	Implication in disease	Therapeutic strategies	Therapeutic effect	Reference
**Chaperoning**					
Hsp70 (*HSPA1A*)	Directs client proteins for degradation by the UPS; serves as a disaggregase against fibrillary aggregates	Component of Lewy bodies in PD	Induction of expression by small molecules (e.g., geldanamycin)^10^; genetic overexpression^1,3,6,8^; induction by cell-penetrating recombinant DJ-1 protein^2^ (*PARK7*), by phenylbutyrate-induced DJ-1 expression^9^, or by CHIP overexpression^6^	Protects against α-syn toxicity in *Drosophila melanogaster* despite the presence of inclusions^1^; reduces insolubility of α-syn in α-syn overexpressing mice^3^; prevents α-syn accumulation and aggregation in human H4 neuroglioma cells^4,5^ and dopaminergic MES cells; in dopaminergic N27 cells, protects against α-syn toxicity^9^; in SK-N-SH cells^7^, prevents oxidative stress, dopamine (DA) loss and cell death^2^	Auluck et al., 2002^1^, 2005^10^; McLean et al., 2002^4^; Klucken et al., 2004^3^; Zhou et al., 2004^8^, 2011^9^; Shin et al., 2005^6^; Yu et al., 2005^7^; Batelli et al., 2008^2^; Outeiro et al., 2008^5^; Duennwald et al., 2012; Gao et al., 2015; Jackrel and Shorter, 2015; Shorter, 2016; Sharma and Priya, 2017
Hsp90 (*HSP90AB 1*)	Stabilizes client proteins by preventing Hsp70-mediated UPS targeting; favors fibrillization of α-syn, shifting the aggregation eqilibrium away from early toxic soluble misfolded species	Component of Lewy bodies in PD, increase in insoluble fraction in temporal cortex from LBD	Inhibition by small molecules (e.g., geldanamycin)^10^	Reduces α-syn aggregation and toxicity in human H4 cells^11^; protects against α-syn toxicity in *D. melanogaster* despite the presence of inclusions^10^	Auluck and Bonini, 2002; Auluck et al., 2005; Cantuti-Castelvetri et al., 2005^11^; Putcha et al., 2010; Gao et al., 2015
Hsp40 (*DNAJB1*)	With Hsp70, targets client proteins for UPS-mediated degradation; participates in α-syn disaggregation together with Hsp110 and Hsp70	Component of Lewy bodies in PD	Overexpression by transfection^4,12^	Reduces α-syn aggregation in human H4 cells^4^; lowers α-syn accumulation and aggregation in SK-N-SH cells^12^	McLean et al., 2002^4^; Fan et al., 2006^12^; Duennwald et al., 2012; Jackrel and Shorter, 2015; Shorter, 2016; Sharma and Priya, 2017
Torsin-1A (*TOR1A*)	Chaperone with ATPase activity, homolog of yeast Hsp104	Component of Lewy bodies and Lewy neurites in LBD	Overexpression by transfection^12^	Reduces α-syn aggregation in human H4 cells	McLean et al., 2002
proSAAS (*PCSK1N*)	Neural-specific secretory chaperone, prevents α-syn aggregation	Component of Lewy bodies in PD	Overexpression using viral vector; extracellular treatment with recombinant proSAAS^13^	Blocks α-synuclein-induced cytotoxicity in primary cultures of nigral dopaminergic neurons and in dopaminergic SH-SY5Y cells^13^	Jarvela et al., 2016^13^
ERdj5 (*DNAJC10*)	Endoplasmic reticulum-resident thioredoxin disulfide reductase, regulates degradation of misfolded proteins via ERAD (endoplasmic-reticulum associated degradation)	Unknown	Overexpression in transgenic *C. elegans*^14^	Protects against α-syn aggregation and toxicity, restoring age-dependent mobility defects and loss of dopaminergic neurons in *Caenorhabditis elegans*^14^	Munoz-Lobato et al., 2014^14^
GRP78 (*HSPA5*)	Endoplasmic reticulum-resident chaperone, induced during the Unfolded Protein Response	Unknown	Recombinant Adeno-Associated Viruses (rAAV)-mediated overexpression in the substantia nigra (SN)	In rats overexpressing α-syn in the substantia nigra, co-overexpression of GRP78 attenuates α-syn-induced dopaminergic neuron loss and motor deficits^15^	Gorbatyuk et al., 2012; Salganik et al., 2015^15^
TRAP-1 (*TRAP1*)	Mitochondrial Hsp75 chaperone with ATPase activity	Unknown	Overexpression^16^	Suppresses α-syn toxicity in mutant A53T α-syn expressing *D. melanogaster*, rat primary neurons and HEK293 cells^16^	Butler et al., 2012^16^
HDAC6 (*HDAC6*)	Histone deacetylase, mediates α-syn degradation by inducing the Heat Shock Response	Component of Lewy bodies in PD	Overexpression by transfection^17^	Decreases α-syn oligomers and toxicity in SK-N-SH cells^17^	Miki et al., 2011; Du et al., 2014^17^
**Ubiquitin-Proteasome System**					
UCHL1 (*UCHL1*)	Ubiquitin carboxy-terminal hydrolase, involved in the processing of ubiquitin precursors and ubuquitinated proteins	Mutated in an autosomal-dominant form of PD, component of LB in sporadic PD	Pharmacological inhibition using LDN-57444^18^	In primary neurons and hippocampal tissue of α-syn overexpressing mice, enhanced synaptic clearance of α-syn^18^	Maraganore et al., 2004; Xia et al., 2008; Cartier et al., 2012^18^; Kumar et al., 2017
CHIP (*STUB1*)	E3 ubiquitin-protein ligase, targets toxic α-syn oligomers toward proteasome- and ALP-mediated degradation	Component of Lewy bodies in PD	Gene therapy (overexpression using a viral vector)^19^	Mediates the degradation of α-syn *in vivo* in rats, but also induces tyrosine hydroxylase degradation, limiting its therapeutic interest^19^	Shin et al., 2005; Tetzlaff et al., 2008; Dimant et al., 2014^19^
USP9X (*USP9X*)	Deubiquitinates α-syn, preventing its degradation by the proteasome	Decreased activity in PD and LBD, component of LB	Overexpression by transfection^20^	Decreases α-syn aggregation and toxicity in SH-SY5Y dopaminergic cells upon proteolytic impairment^20^	Rott et al., 2011^20^
**Macroautophagy**					
mTOR (*MTOR*)	Serine/Threonine kinase which acts as an autophagy repressor	Decreased activity in patient-derived GBA mutant fibroblasts	Inhibition using rapamycin^21,22^	Autophagic clearance of α-syn, protection of DA neurons and improvement in motor function in rodents^21,22^ (with possible adverse effects)	Kahan, 2011; Decressac et al., 2013^21^; Magalhaes et al., 2016; Tian et al., 2016^22^
SIRT2 (*SIRT2*)	Deacetylates α-syn on lysines 6 and 10	Unknown	Knock-out in mice^23^	In mice, protects against DA neurons loss caused by overexpression of α-syn in the SN, or by MPTP injection^23^	de Oliveira et al., 2017^23^
PLK2 (*PLK2*)	Phosphorylates α-syn at S129 to stimulate its removal by autophagy	Upregulated in LBD- affected brains	AAV-mediated overexpression in the SN^24^	Reduces α-syn accumulation, DA neurons loss and motor deficits in a rat genetic model of PD^24^	Mbefo et al., 2010; Oueslati et al., 2013^24^; Dahmene et al., 2017
Beclin-1 (*BECN1*)	Regulates the PI3K complex, stimulating autophagosome formation	Unknown	Overexpression (lentivirus)^25^	Reduces α-syn accumulation, ALP defects and neuronal pathology in α-syn transgenic mice^25^	Spencer et al., 2009^25^
Spermidine	Activates autophagy, counteracting age-assocaited cell death	Unknown	Spermidine administration^26^	Rescues α-syn toxicity, motor deficits and loss of DA neurons in *C. elegans* and *D. melanogaster^26^*	Büttner et al., 2014^26^
**Chaperone-mediated autophagy**					
Lamp2A (*LAMP2*)	CMA receptor, rate-limiting factor of CMA. Translocates α-syn into lysosomes for degradation	Decreased levels correlate with α-syn accumulation in PD	Overexpression using AAV or rAAV^27^	Upregulates CMA activity, reducing α-syn levels and α-syn toxicity in SH-SY5Y DA cells, rat primary cortical and nigral DA neurons^27^	Xilouri et al., 2013^27^; Murphy et al., 2014; Xilouri et al., 2016

*Targets: main PQC pathways and biological targets with therapeutic potential. Physiologic function: function of the target with respect to α-syn–relevant biological pathways. Implication in disease: pathologic evidence for the implication of the target in patients with synucleinopathies. Therapeutic strategies: describes experimental strategies used to manipulate a given target. Therapeutic effect: describes biologic effects on α-syn pathology observed upon application of the corresponding therapeutic strategies. Superscript numbers indicate the corresponding references for each model. The corresponding human Gene Symbol related to proteins of interest is indicated in parentheses*.

### Ubiquitin-Proteasome System

Dysfunction in the UPS contributes to α-syn pathology with proteasomal subunits and ubiquitinated α-syn observed in LBs (Ii et al., 1997; Hasegawa et al., 2002). In sporadic PD, 20S and 26S proteasome activity are reduced, and α-syn aggregates can inhibit proteasome function (Bence et al., 2001; Tanaka et al., 2001). Many dysfunctions in Ub ligases (specifically E3s) have been linked to α-syn quality control. Indeed, various E3s have been identified in LBs, including CHIP (**Table 1**), E6AP and SIAH (Schlossmacher et al., 2002; Liani et al., 2004; Shin et al., 2005; Mulherkar et al., 2009). CHIP, a co-chaperone with E3 Ub ligase activity, regulates α-syn proteasomal degradation, in collaboration with Hsc70, Hsp70, and Hsp90. Like CHIP, E6AP triggers α-syn degradation while SIAH monoubiquitinates α-syn to promote its aggregation (Rott et al., 2008; Mulherkar et al., 2009). Mutations in the gene encoding Parkin E3 Ub ligase are responsible for inherited PD (Olzmann et al., 2007; Lonskaya et al., 2013). Although these loss-of-function mutations are associated with PD, they do not lead to LB pathology (Shimura et al., 2000). It has been suggested that Parkin K63-linked polyubiquitination favors LB formation (Olanow et al., 2004; Kramer and Schulz-Schaeffer, 2007; Olzmann and Chin, 2008), and deficient Parkin activity would favor accumulation of earlier, potentially toxic aggregation intermediates. However, this awaits further confirmation, and to date, the role of Parkin Ub-ligase activity in mitochondrial quality control (Roberts et al., 2016) appears more relevant to PD pathogenesis than in LB formation.

Since proteasomes can degrade α-syn (Bennett et al., 1999), and regulation of α-syn ubiquitination has been implicated in PD (Liani et al., 2004; Rott et al., 2008, 2011), enhancing UPS activity could stimulate α-syn degradation and reduce aggregation-linked pathology (Opattova et al., 2015). Non-aggregated α-syn could be specifically targeted to the proteasome, thereby preventing aggregated α-syn from further inhibiting proteasome catalytic activity (Stefanis et al., 2001; Snyder et al., 2003; Chen et al., 2006). Selective enhancement of α-syn targeting to proteasomes is a more desirable approach to broader enhancement of UPS activity, which may lead to serious adverse effects. This could be achieved by increasing the activity of the specific machinery that controls the ubiquitination of α-syn, such as the druggable deubiquitinase USP9X (Rott et al., 2011, **Table 1**), although only inhibitors have been reported so far (Peterson et al., 2015). Deubiquitination of α-syn might redirect the α-syn burden toward the ALP, which is generally recognized as a more efficient α-syn degradation pathway than the UPS (Vogiatzi et al., 2008). It should be noted that α-syn ubiquitination can serve as a signal for lysosome-dependent degradation (Tofaris et al., 2011; Braun, 2015; Alexopoulou et al., 2016), illustrating a complex cross-talk between post-translational modifications of α-syn and cellular degradation machineries (Choi et al., 2012; Haj-Yahya et al., 2013; Shahpasandzadeh et al., 2014; Tenreiro et al., 2014; de Oliveira et al., 2017). It remains unclear which α-syn degradation pathway is favored, therefore further study is needed before a viable therapeutic strategy can be designed to enhance UPS-mediated α-syn degradation.

### Autophagy-Lysosome Pathway

The ALP is thought to be the most efficient pathway for degradation of α-syn (Vogiatzi et al., 2008), with dysfunction causing accumulation and aggregation of α-syn. Defects in the ALP have been linked with an increasing number of genetic variants identified as causative or associated with PD risk (Gan-Or et al., 2015), including Vps35, a component of the retromer that mediates retrograde transport from endosomes to Golgi, the lysosomal ATPase pump ATP13A2, and LRRK2 (Ramirez et al., 2006; Usenovic et al., 2012; Orenstein et al., 2013; Kong et al., 2014; Tsunemi and Krainc, 2014; Tang et al., 2015; Follett et al., 2016). Polymorphisms in genes encoding lysosomal enzymes, acid sphingomyelinase (*SMPD1* gene), and β-glucocerebrosidase (GBA, *GBA1* gene), are also risk factors for synucleinopathies (Neumann et al., 2009; Dagan et al., 2015; Gan-Or et al., 2015). A reduction in GBA expression and activity is observed in the substantia nigra and cerebellum of patients with sporadic PD (Gegg et al., 2012), and the inhibition of GBA or its transporter Limp2 is sufficient to stimulate α-syn aggregation through autophagic inhibition (Rothaug et al., 2014; Du et al., 2015). Polymorphisms in the lysosomal K^+^ channel encoding gene *TMEM175* are risk factors for PD (Nalls et al., 2014). TMEM175 deficiency causess ALP dysfunction and increased α-syn aggregation (Jinn et al., 2017). Additional ALP-related genes were just recently linked to PD (Chang et al., 2017), converging into a unifying theory for PD pathogenesis, where the ALP is challenged by defects in synaptic exocytosis, endocytosis, and endosomal trafficking, resulting in neuron dysfunction and death (Trinh and Farrer, 2013).

Macroautophagy is responsible for degrading most of the aggregated, proteasome-resistant, α-syn, and enhancing this process represents a promising therapeutic strategy (**Figure 1** and **Table 1**). The mTOR inhibitor rapamycin activates macroautophagy, prevents α-syn accumulation and aggregation, and ameliorates motor symptoms, but adverse effects have been reported (Kahan, 2011; Li et al., 2014; Tian et al., 2016). More recently, it was shown that acetylation of α-syn increases macroautophagy-mediated degradation of α-syn aggregates, with knock-out of the α-syn deacetylase SIRT2 protects against α-syn–induced dopaminergic cell loss *in vivo* (de Oliveira et al., 2017, see **Table 1**). Independent from UPS-targeting, modulation by the deubiquitinase USP8 and the Ub-ligase Nedd4 of α-syn modification by K63-linked Ub appears to control its autophagic degradation (Braun, 2015; Alexopoulou et al., 2016). A better understanding of the specific effects of various post-translational modifications will be necessary to appropriately modulate α-syn clearance by macroautophagy.

CMA specifically degrades physiologic α-syn (which contains a KFERQ-like motif, VKKDQ), whereas pathologic α-syn inhibits CMA, thus enhancing aggregation of itself and other LB components (Martinez-Vicente et al., 2008; Vogiatzi et al., 2008; Xilouri et al., 2009). Accordingly, overexpression of certain PD-associated microRNAs is suspected to be responsible for pathologic CMA downregulation through decreased Hsc70 and Lamp2A expression. This correlates with α-syn accumulation in brains of patients with PD (Alvarez-Erviti et al., 2013; Murphy et al., 2015). CMA-mediated degradation of α-syn and LRRK2 is also impaired by mutants of these proteins that cause inherited PD (A53T and A30P α-syn mutants; G2019S and R1441C LRRK2 mutants). These mutants are recognized by Hsc70 and targeted to the lysosomal membrane, but fail to be translocated into the lysosome due to an aberrantly high affinity for Lamp2A. This impairs CMA-mediated degradation of these proteins and CMA activity, contributing to PD pathology (Cuervo et al., 2004; Orenstein et al., 2013). Deficiencies in CMA (caused by *LRRK2* or *VPS35* PD-associated mutations for example, Orenstein et al., 2013; Tang et al., 2015; Ho et al., 2016) cause accumulation of α-syn, favoring the emergence of aberrant α-syn species that hinder the function of the Lamp2A receptor. Lamp2A overexpression efficiently prevents α-syn burden in cellular and animal models of PD and counteracts motor deficits (see **Table 1**). Whether this strategy can reverse α-syn pathology in a clinical context, in a safe and effective way, still needs to be determined, especially since CMA cannot mediate the degradation of aggregated species. The role of CMA in PD pathogenesis has been reviewed recently (Sala et al., 2016), and will not be discussed further here. Notably, strategies aiming at activating both macroautophagy and CMA are also being explored (Gan et al., 2012; Lastres-Becker et al., 2016), such as overexpression of the transcription factor NRF2, which protects against α-syn pathology and increases its turnover through unknown ALP-dependent mechanisms (Skibinski et al., 2017).

A prerequisite for efficient α-syn degradation by ALP is the adequate function and hydrolytic capacity of lysosomes. Improper activity of lysosomal hydrolases, due to mutations, sorting defects, or altered lysosomal homeostasis, has emerged as a critical step in the development of PD (Trinh and Farrer, 2013; Gan-Or et al., 2015) and other synucleinopathies, particularly those associated with lysosomal storage disorders (Gaucher disease, Niemann-Pick disease; Cullen et al., 2011; Osellame and Duchen, 2013; Osellame et al., 2013). Stimulating lysosome biogenesis via activation of ALP transcriptional regulator TFEB improves autophagic α-syn clearance (Decressac et al., 2013; Kilpatrick et al., 2015), and reduces α-syn toxicity in rats (Decressac et al., 2013). Enhancing lysosomal hydrolase activity also improves α-syn degradation by the ALP, as shown for the α-syn-cleaving proteases cathepsins D, B and L (Miura et al., 2014; Lehri-Boufala et al., 2015; McGlinchey and Lee, 2015) or GBA (Yang et al., 2017). Finally, rescue of lysosomal hydrolase misfolding by small molecule chaperones (Sanchez-Martinez et al., 2016; Yang et al., 2017) or enhancement of endo-lysosomal trafficking of ALP components (Chung et al., 2013; Miura et al., 2014; Tang et al., 2015) are likely to mitigate α-syn pathology.

### Other PQC Mechanisms

Other PQC mechanisms exist that are less commonly referred to in the context of α-syn pathology. α-syn synthesis could be reduced in the first place to prevent its accumulation. Several microRNAs target α-syn mRNA to reduce its expression in cell culture and *in vivo* (Junn et al., 2009; Doxakis, 2010; Singh and Sen, 2017). The therapeutic potential of this mechanism remains to be evaluated, especially regarding potential adverse effects of a lack of functional α-syn on the dopamine system (Abeliovich et al., 2000). Finally, unconventional secretion of misfolded proteins (misfolding-associated protein secretion, MAPS) was recently suggested to protect individual cells from misfolded proteins by delivering them to the extracellular space (Lee et al., 2016). In the context of a multicellular organism, however, this secretion might be harmful by contributing to the prion-like spreading of misfolded proteins including α-syn.

## Concluding Remarks

Extensive genetic and experimental evidence indicate that PQC deficiencies influence the development of synucleinopathies. Despite the potential of several experimental strategies targeting PQC to attenuate α-syn pathology, translation into therapy is still pending. Whether these approaches will be clinically effective, where synuclein pathology is pre-existant, remains unknown. No successful clinical trial has been reported for synucleinopathies, but targeting PQC bears great promise as such strategies have proven effective to treat diseases such as cystic fibrosis or cancer (Teicher and Tomaszewski, 2015; Hegde et al., 2017). Further functional characterization of genes associated with synucleinopathies, will provide important insights regarding the molecular mechanisms that can be targeted to enhance PQC function, boost α-syn degradation, and prevent its aggregation. For patients with familial synucleinopathies, the upcoming era of personalized medicine, including the use of patient-derived induced pluripotent-stem cells and genome-editing, might allow correction of patient-specific mutations or PQC impairments. However, in sporadic cases, where genetic contributions are unknown (the majority of PD cases), simultaneous enhancement of several components of PQC machinery will likely be necessary to stop the progression, or even reverse the course, of these devastating neurodegenerative diseases.

## Author Contributions

D-LM, BV, and TD: conception and organization of content of the mini-review. D-LM: design and generation of **Figure 1**, writing of introduction, and sections on α-syn pathology and defective PQC in synucleinopathies. BV: design and generation of **Table 1**, writing of section on therapeutic strategies, the abstract and concluding remarks, and assembly of manuscript. EF: overall revision. TD: in-depth editing of manuscript, and overall revision.

## Conflict of Interest Statement

The authors declare that the research was conducted in the absence of any commercial or financial relationships that could be construed as a potential conflict of interest.

## Abbreviations

α-syn: α-synuclein
ALP: autophagy-lysosome pathway
CHIP: carboxyl terminus of Hsp70-interacting protein
CMA: chaperone-mediated autophagy
E6AP: E6 associated protein
GBA: β-glucocerebrosidase
Hsc: heat shock cognate
Hsp: heat shock protein
LB: Lewy bodies
LBD: Lewy body dementia
MPTP: 1-methyl-4-phenyl-1,2,3,6-tetrahydropyridine
MSA: multiple system atrophy
NAC: non-amyloid-β component
PD: Parkinson’s disease
PQC: protein quality control
SIAH: seven in absentia homolog
Ub: ubiquitin
UPS: ubiquitin-proteasome system.
